# Supplementary material for: The Lithuanian Lung Cancer Screening Model: Results of a Pilot Study
Source: Cancers (Basel). 2025 Jun 12;17(12):1956. doi: 10.3390/cancers17121956 (PMC12191307; doi:10.3390/cancers17121956)
Supplement: Supplementary file 1 [file cancers-17-01956-s001.zip › Table S1.pdf]

cancers

MDPI

**Table S1.** Newly Diagnosed Morbidities.

| ICD-10-AM | Diagnosis according to ICD-10-AM | Number (% of all participants) |
|---|---|---|
| | **Respiratory System Diseases** | |
| D38.3 | Neoplasm of uncertain or unknown behavior of mediastinum | 2 (0.2%) |
| J20.9 | Acute bronchitis, unspecified | 1 (0.1%) |
| J21.9 | Acute bronchiolitis, unspecified | 2 (0.2%) |
| J41.0 | Simple chronic bronchitis | 1 (0.1%) |
| J43 | Emphysema | 15 (1.5%) |
| J47 | Bronchiectasis | 10 (1.0%) |
| J84.9 | Interstitial pulmonary disease, unspecified | 11 (1.1%) |
| J94.9 | Pleural condition, unspecified | 1 (0.1%) |
| J98.4 | Other disorders of the lung | 3 (0.3%) |
| J98.5 | Diseases of mediastinum not elsewhere classified | 4 (0.4%) |
| J98.8 | Other specified respiratory disorders | 1 (0.1%) |
| Q33.2 | Pulmonary sequestration | 1 (0.1%) |
| | **Cardiovascular Diseases** | |
| I25.11 | Atherosclerotic heart disease of native coronary artery | 165 (16.3%) |
| I27.0 | Primary pulmonary hypertension | 1 (0.1%) |
| I31.3 | Pericardial effusion, noninflammatory | 1 (0.1%) |
| I31.9 | Pericardial disease, unspecified | 1 (0.1%) |
| I34.9 | Nonrheumatic mitral valve disorder, unspecified | 1 (0.1%) |
| I35.9 | Aortic valve disorder, unspecified | 1 (0.1%) |
| I70.0 | Atherosclerosis of aorta | 5 (0.5%) |
| I70.8 | Atherosclerosis of other arteries | 6 (0.6%) |
| I71.2 | Thoracic aortic aneurysm, without rupture | 6 (0.6%) |
| I71.4 | Abdominal aortic aneurysm, without rupture | 1 (0.1%) |
| I71.6 | Thoracoabdominal aortic aneurysm, without rupture | 1 (0.1%) |
| I71.9 | Aortic aneurysm, unspecified site, without rupture | 1 (0.1%) |
| | **Other Conditions** | |
| D21 | Benign neoplasm of connective and other soft tissue | 1 (0.1%) |
| D44.1 | Neoplasm of uncertain behavior of adrenal gland | 1 (0.1%) |
| D73.8 | Other specified diseases of spleen | 1 (0.1%) |
| E04.9 | Nontoxic goiter, unspecified | 3 (0.3%) |
| K22.8 | Other specified diseases of esophagus | 1 (0.1%) |
| K44 | Diaphragmatic hernia | 4 (0.4%) |
| K76 | Other liver diseases | 2 (0.2%) |
| M81 | Osteoporosis without pathological fracture | 1 (0.1%) |
| M84.98 | Other specified disorders of bone integrity, unspecified site | 1 (0.1%) |
| M95.4 | Acquired deformity of chest and rib | 1 (0.1%) |
| N13.3 | Other and unspecified hydronephrosis | 1 (0.1%) |

 https://doi.org/10.3390/cancers17121956

| | | |
|---|---|---|
| N20.0 | Calculus of kidney | 1 (0.1%) |
| N60.9 | Benign mammary dysplasia, unspecified | 1 (0.1%) |
| R93.3 | Abnormal findings on imaging of other parts of the digestive tract | 1 (0.1%) |
| S22.02 | Fracture of thoracic vertebra, T3 and T4 level | 1 (0.1%) |
| | **Total newly diagnosed morbidities** | **263** |

2
