# Supplementary figures and images for: The Lithuanian Lung Cancer Screening Model: Results of a Pilot Study
Source: Cancers (Basel). 2025 Jun 12;17(12):1956. doi: 10.3390/cancers17121956 (PMC12191307; doi:10.3390/cancers17121956)

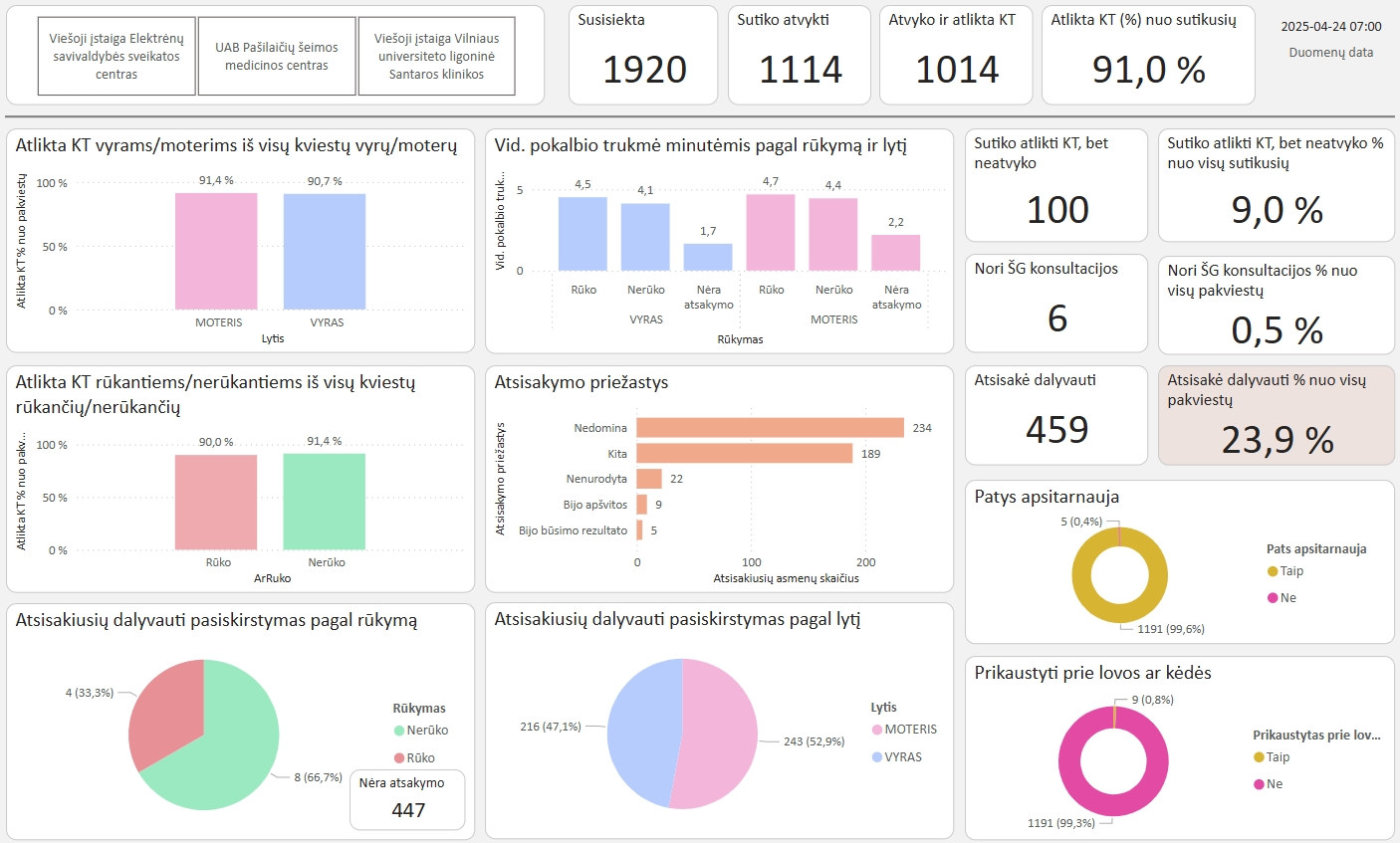

Supplement: Supplementary file 1 [file cancers-17-01956-s001.zip › Figure S1 a.jpg]

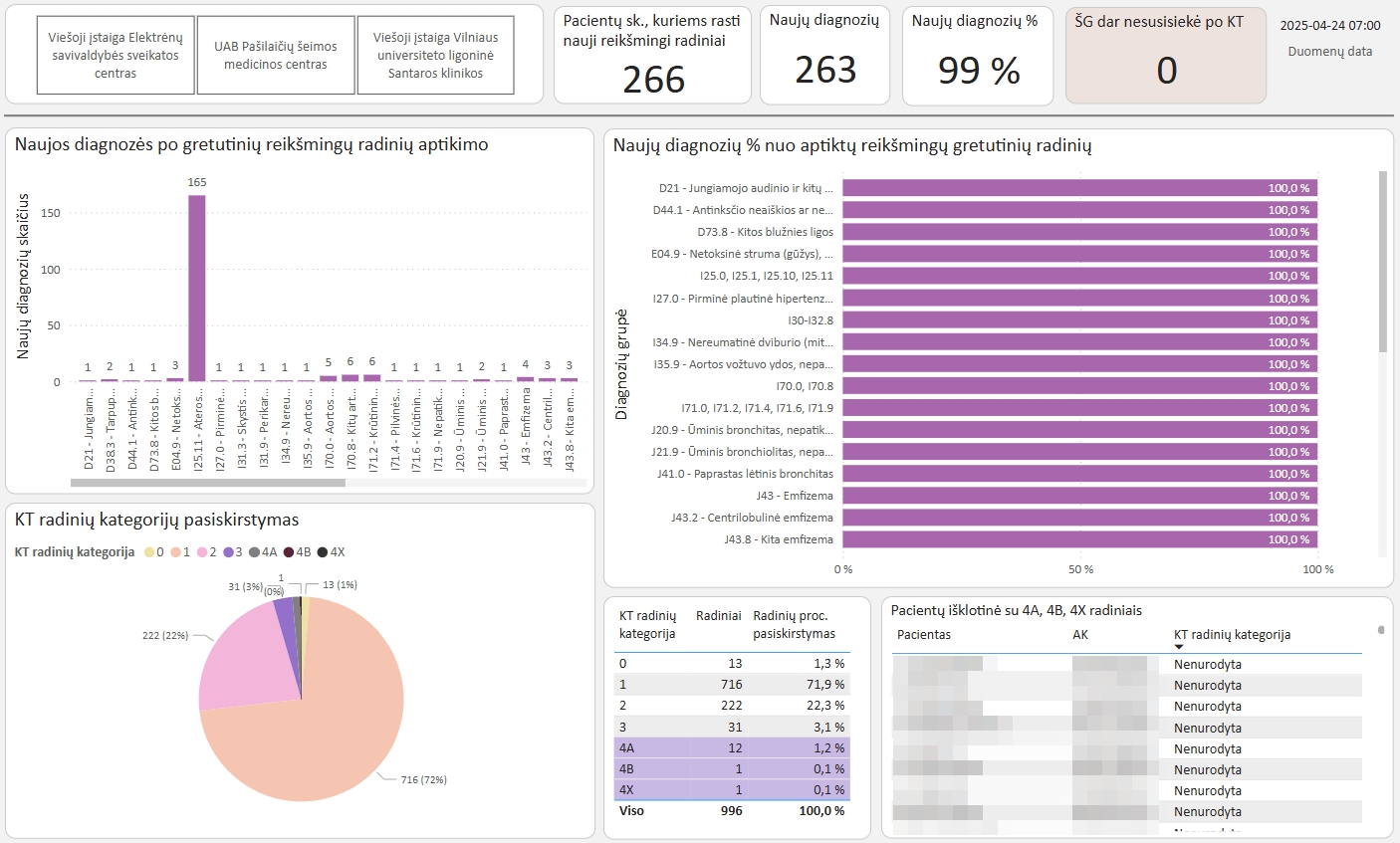

Supplement: Supplementary file 1 [file cancers-17-01956-s001.zip › Figure S1 b.jpg]

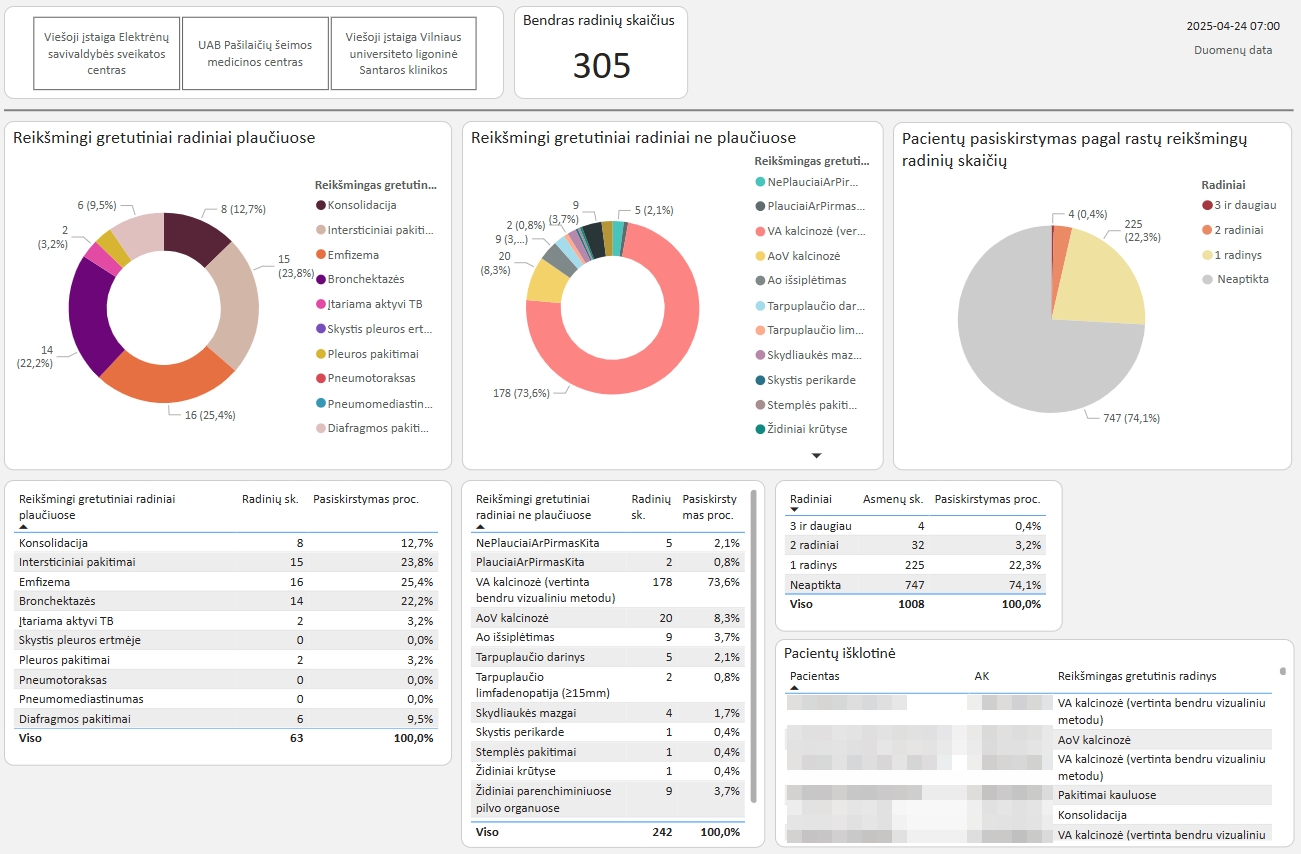

Supplement: Supplementary file 1 [file cancers-17-01956-s001.zip › Figure S1 c.jpg]

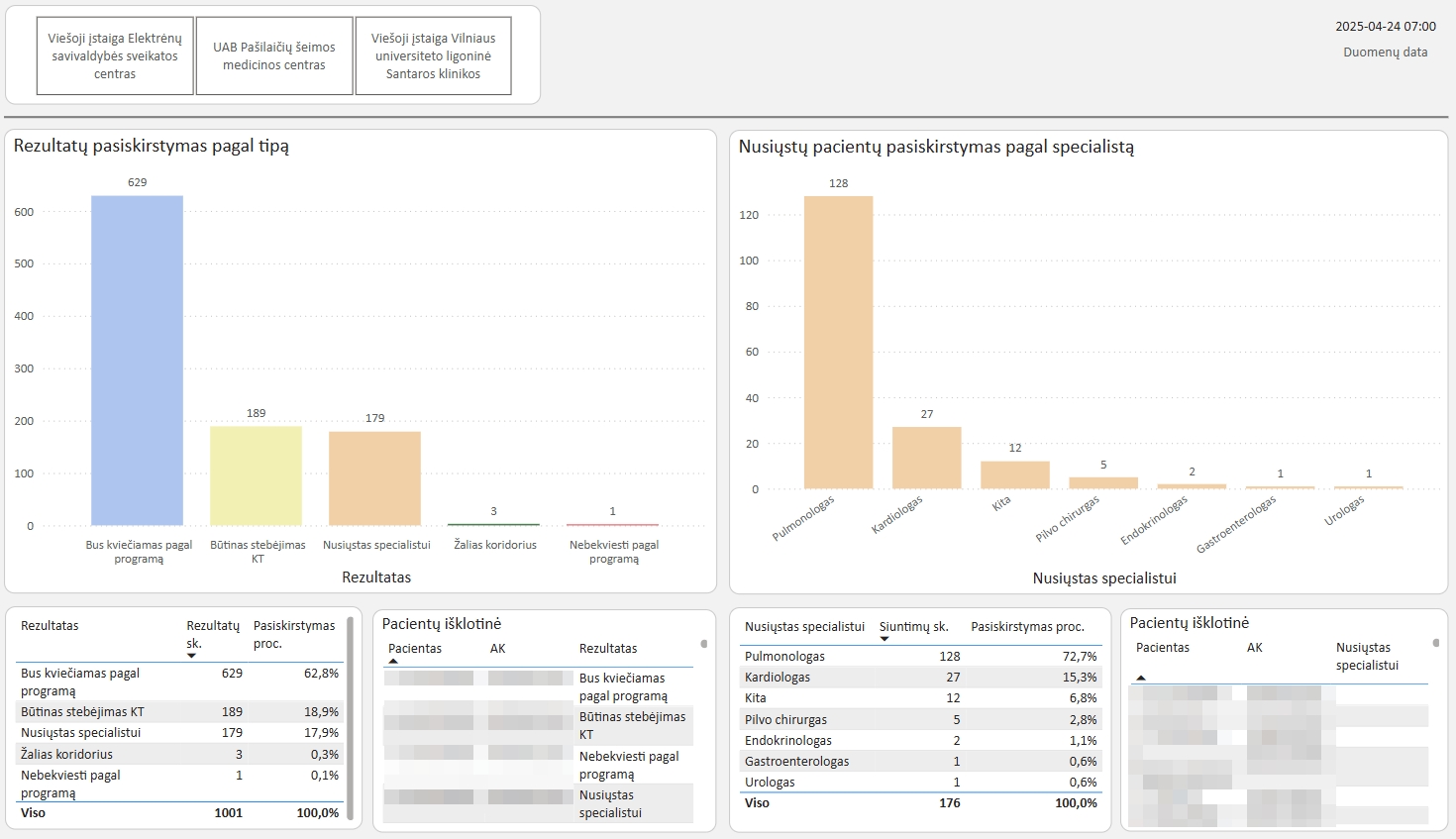

Supplement: Supplementary file 1 [file cancers-17-01956-s001.zip › Figure S1 d.jpg]

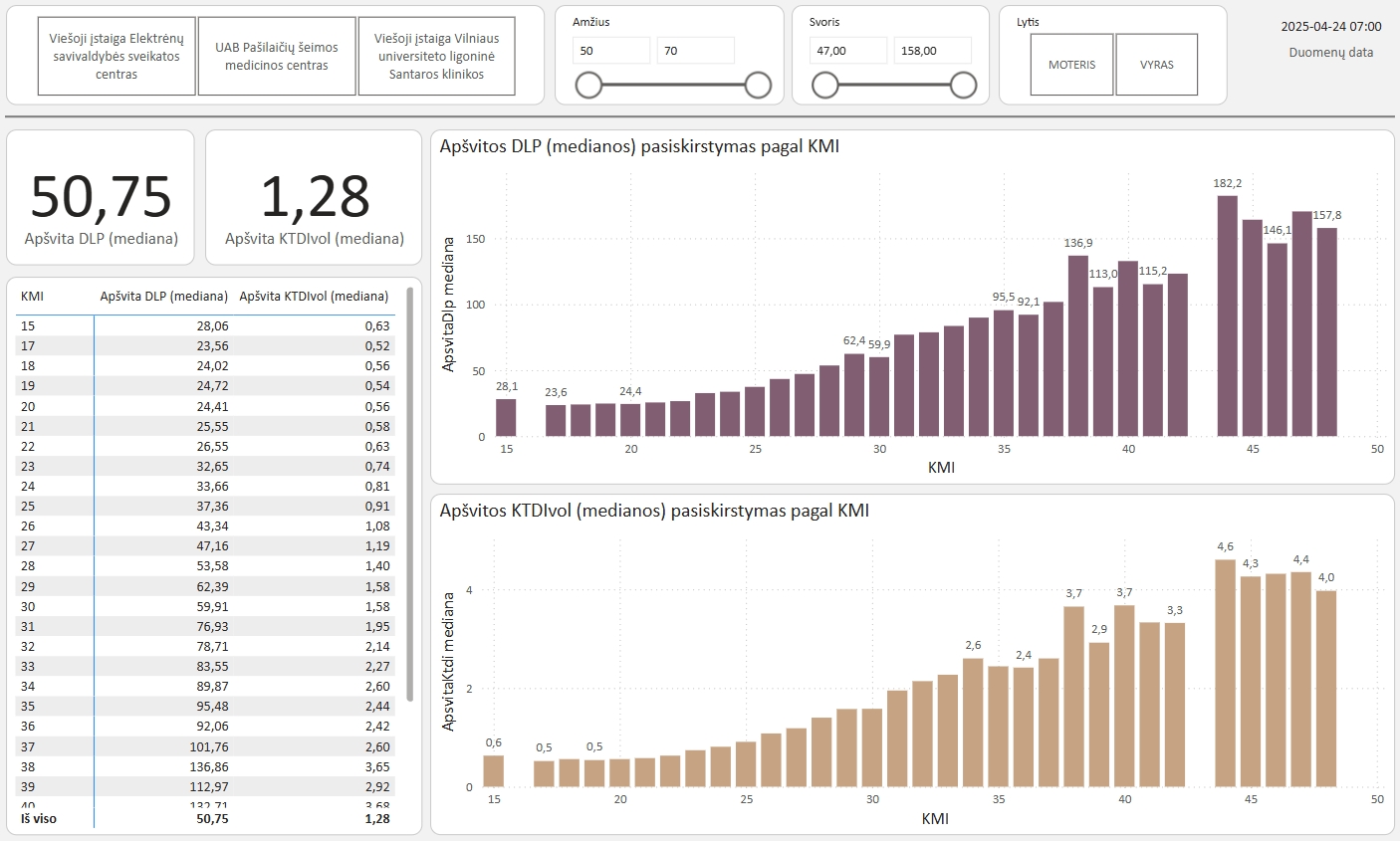

Supplement: Supplementary file 1 [file cancers-17-01956-s001.zip › Figure S1 e.jpg]
